# Supplementary material for: Importance of between and within Subject Variability in Extracellular Vesicle Abundance and Cargo when Performing Biomarker Analyses
Source: Cells. 2021 Feb 24;10(3):485. doi: 10.3390/cells10030485 (PMC7996254; doi:10.3390/cells10030485)
Supplement: Supplementary file 1 [file cells-10-00485-s001.zip › Supplementary data/Supplementary Figures Cells .pdf]

Supplementary Information

Importance of between and within subject variability in small extracellular vesicle abundance and cargo when performing biomarker analyses

Lauren A Newman [1], Alia Famhy [1], Michael J Sorich [1], Oliver G Best [1], Andrew Rowland [1] and Zivile Useckaite [1,*]

Affiliation: [1]College of Medicine and Public Health, Flinders University, Adelaide, SA, Australia

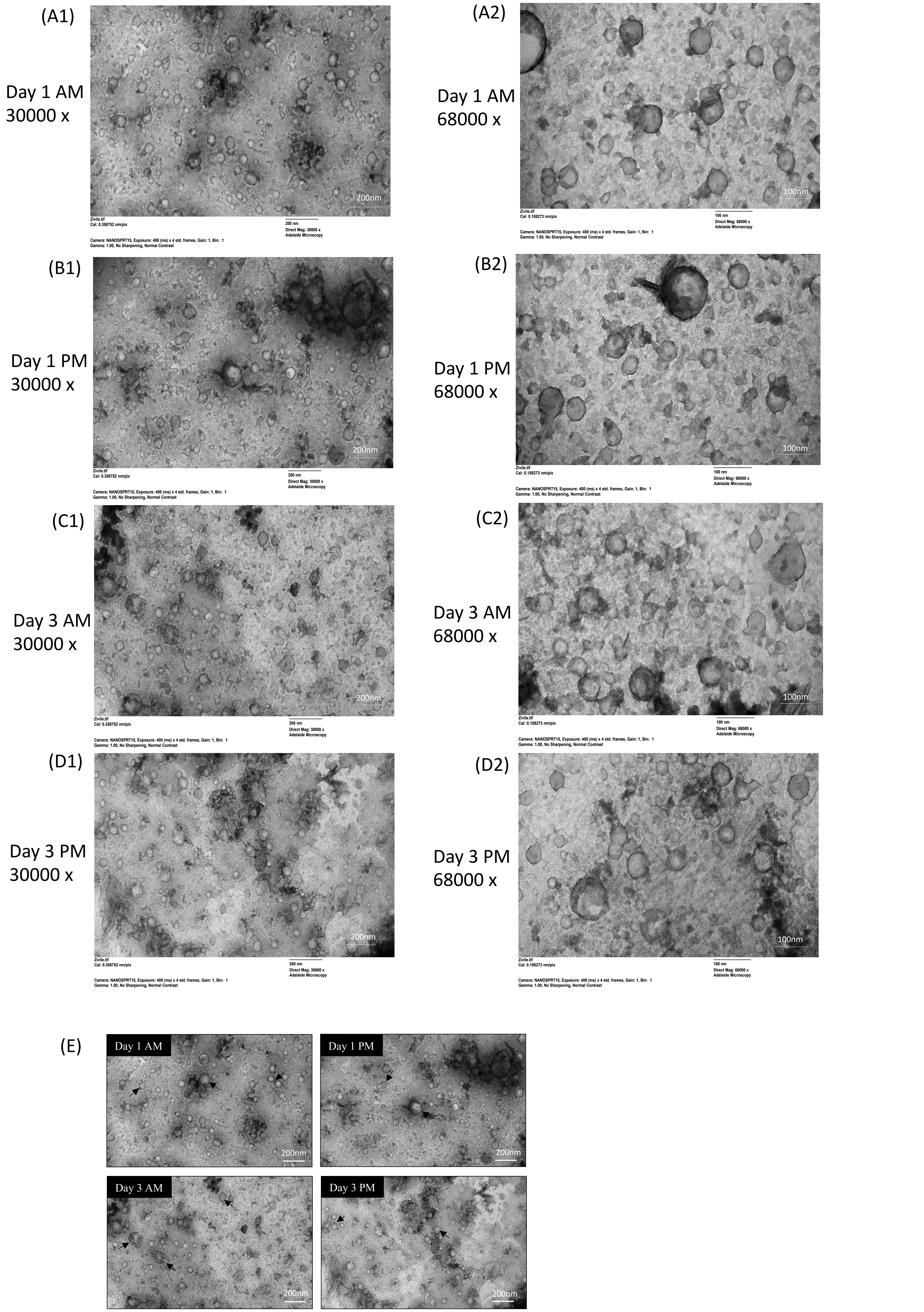

Figure S1. Unedited images for TEM analysis. (A1, B1, C1, D1) magnification of 30000 x, (A2, B2, C2 and D2) magnification of 68000 x. (E) Edited TEM images as presented in the main text (no sharpening, normal contrast).

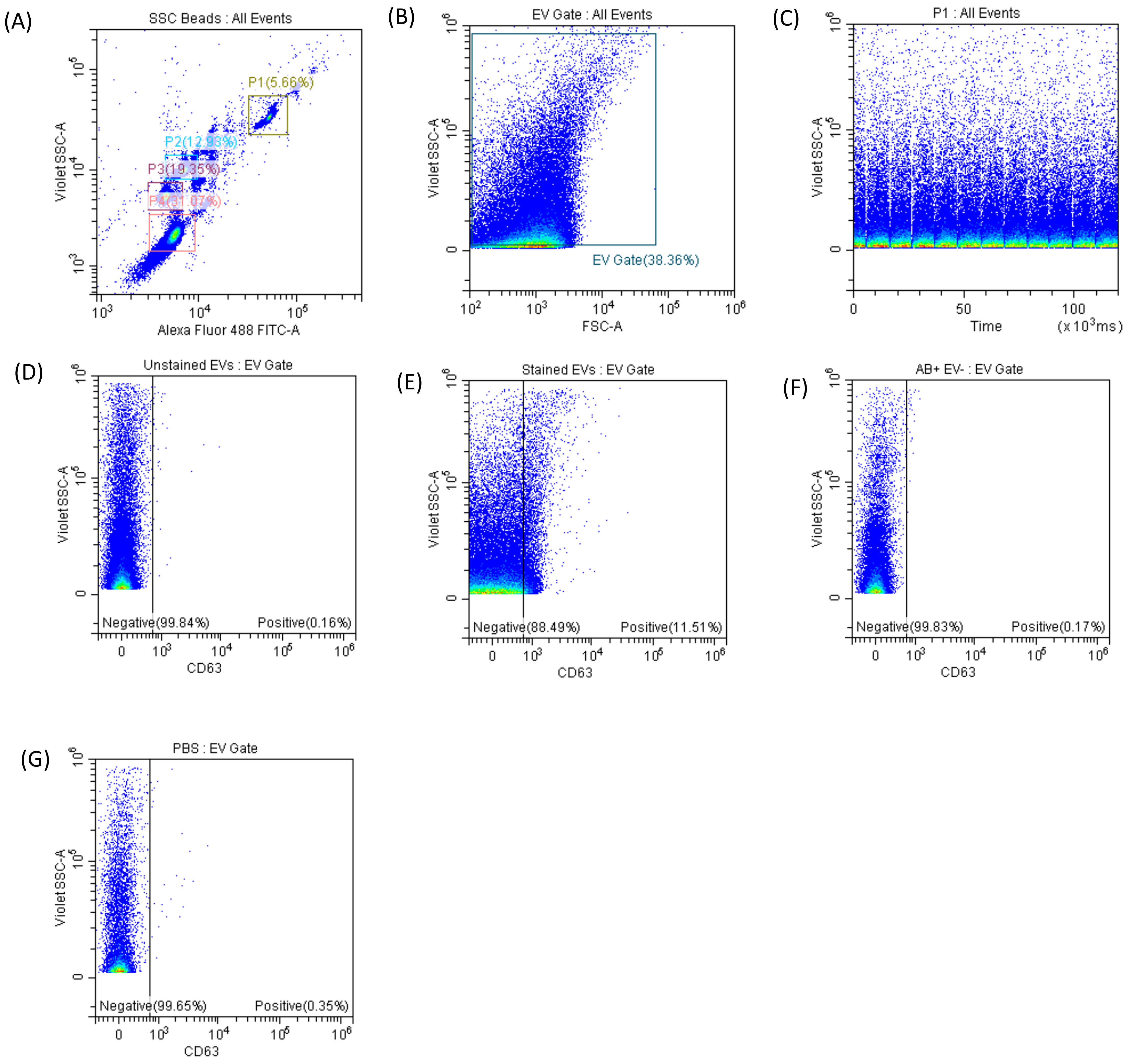

Figure S2. Gating strategy for EV analysis by flow cytometry. (A) Megamix-Plus fluorescent beads in the range 100 – 900nm were identified based on their fluorescent and light scatter properties. Regions were set as P1, P2, P3 and P4 according to the manufacturers recommendations (BioCytex). (B) EVs were gated based on Megamix-Plus beads in (A). (C) Data acquisition was performed using a constant flow rate and monitored using a VSSC *vs* time plot (D) Unstained EV fraction and (E) EV fraction stained for CD63. The percent positive cells and MFI values were calculated relative to unstained controls. (F) Antibody in PBS and (G) PBS alone controls.

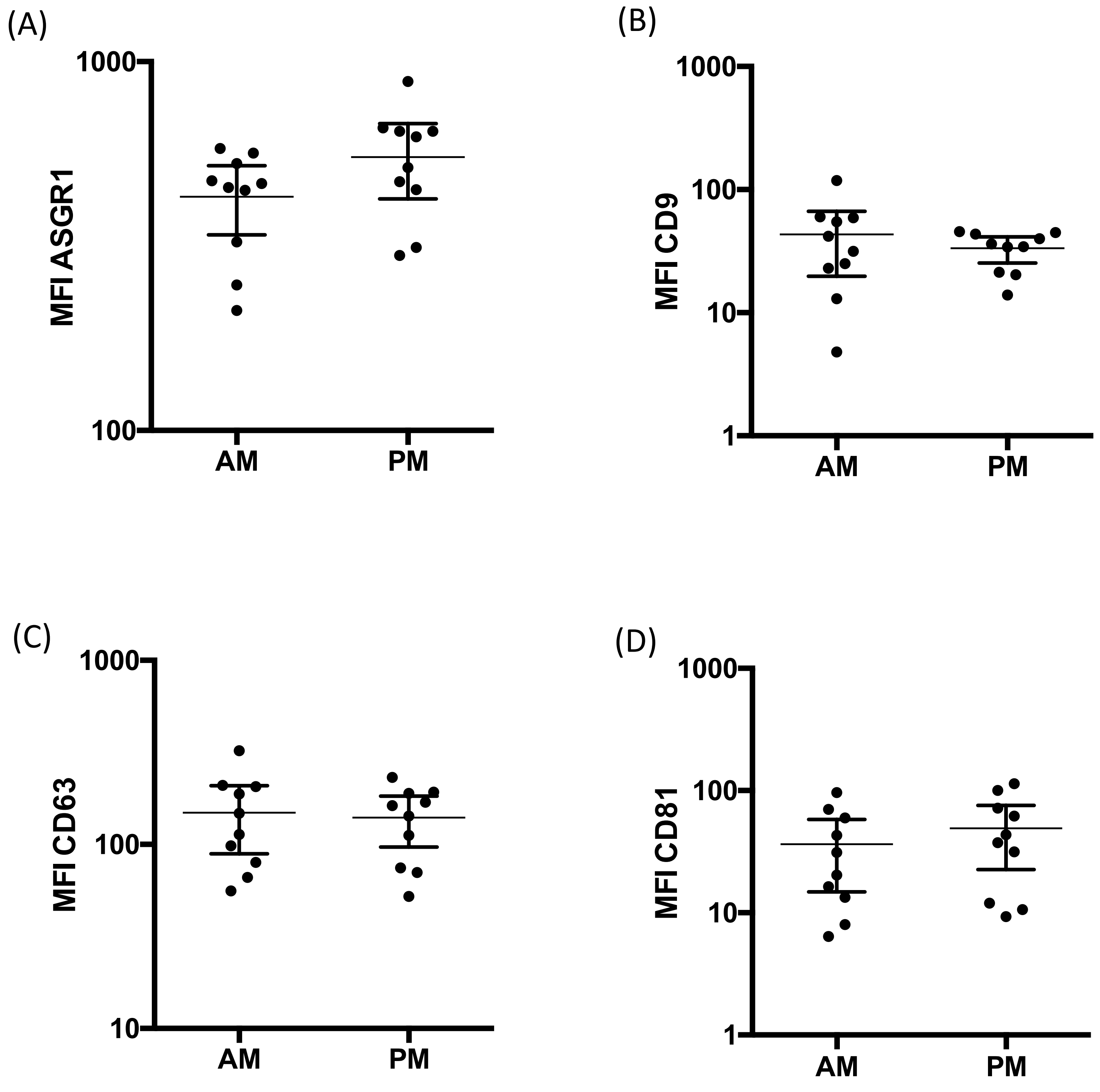

(E)

	MFI ASGR1				MFI CD9			
	AM	PM	diff	p	AM	PM	diff	p
Mean	430.60	551.60			43.17	33.41		
95% CI lower	339.00	424.40	ns	0.0978	19.75	25.40	ns	0.6706
95% CI upper	522.20	678.90			66.90	41.42		
Range	370.60	585.50			114.00	31.70		
	**MFI CD63**				**MFI CD81**			
	AM	PM	diff	p	AM	PM	diff	p
Mean	148.80	139.80			36.46	49.22		
95% CI lower	89.03	96.74	ns	0.785	14.84	22.64	ns	0.411
95% CI upper	208.50	182.80			58.08	75.80		
Range	266.80	179.10			89.90	104.80		

Figure S3. Flow cytometry analysis establishing normal ranges of variability on marker abundances. (A-D) EV surface marker expression presented as MFI, in the morning (AM) and afternoon (PM) of study day 1 in healthy volunteers (n=10). Statistical analysis used paired t-test, Error bars represent mean with 95% CI. (E) Summary data table of A-D.

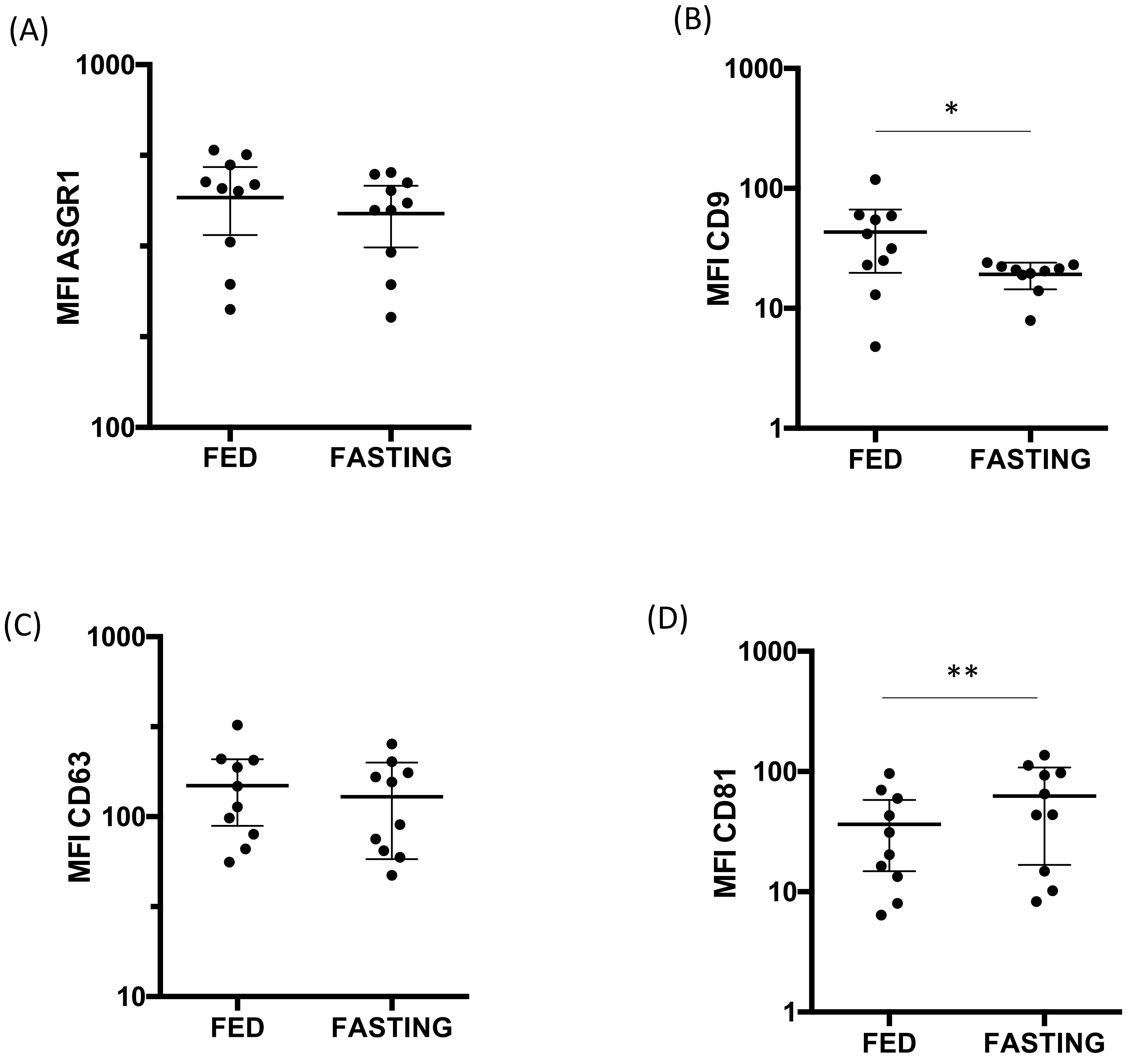

(E)

	MFI ASGR1				MFI CD9			
	FED	FASTING	diff	p	FED	FASTING	diff	p
Mean	430.60	388.80			43.17	19.26		
95% CI lower	339.00	313.70	ns	0.202	19.75	15.79	*	0.049
95% CI upper	522.20	463.90			66.59	22.73		
Range	370.60	302.90			114.00	16.10		
	**MFI CD63**				**MFI CD81**			
	FED	FASTING	diff	p	FED	FASTING	diff	p
Mean	148.80	129.10			36.46	62.59		
95% CI lower	89.03	78.28	ns	0.531	14.84	29.82	**	0.002
95% CI upper	208.50	179.80			58.08	95.36		
Range	266.80	206.80			89.90	128.70		

Figure S4. Flow cytometry analysis investigating the impact of fasting on EV marker abundances. (A-D) EV surface marker expression presented as MFI in serum EVs from fed and fasted subjects(n=10). Statistical analysis used paired t-test, Error bars represent mean with 95% CI. (E) Summary data table of A-D.

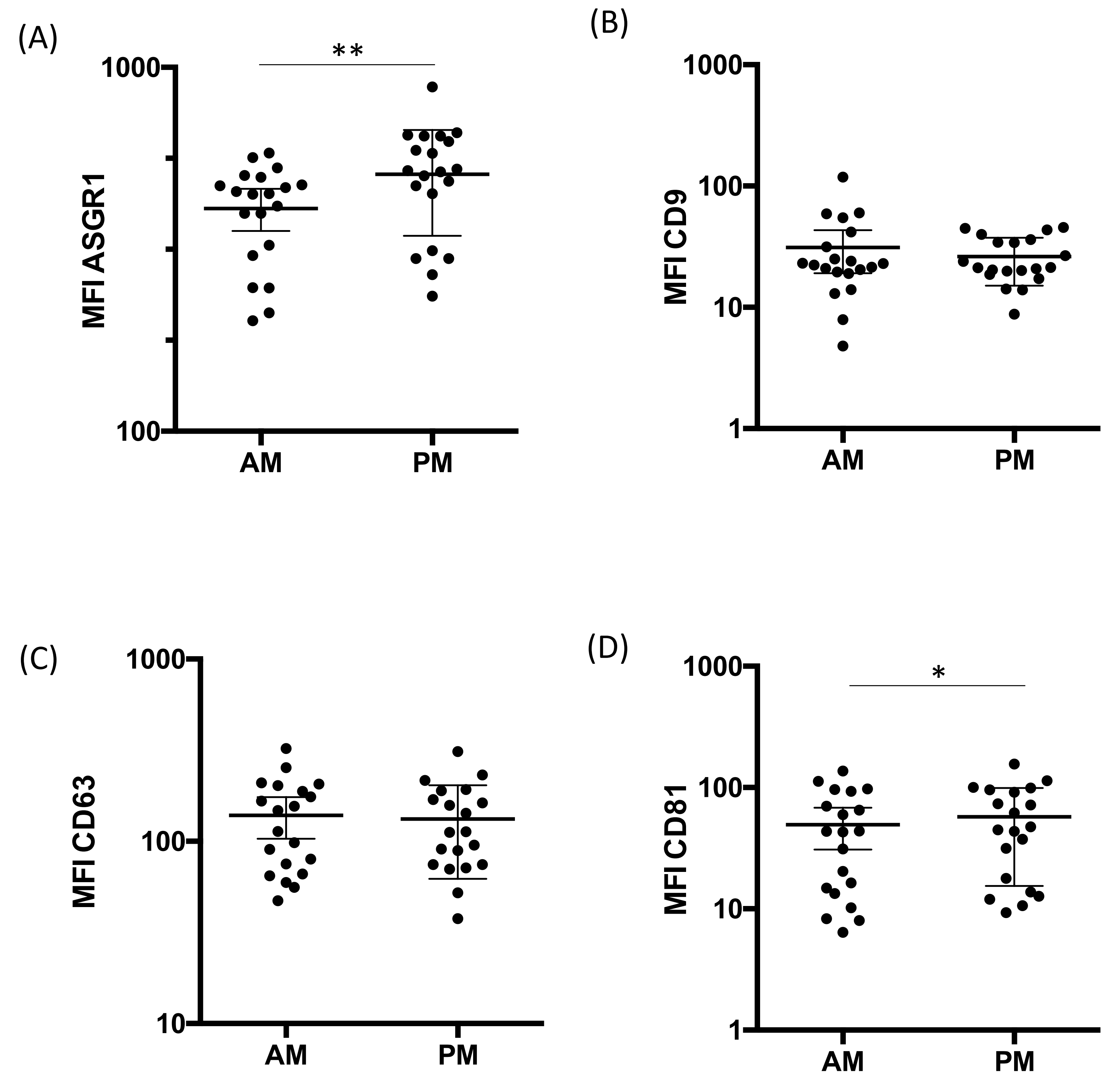

(E)

	MFI ASGR1				MFI CD9			
	AM	PM	diff	p	AM	PM	diff	p
Mean	409.70	508.90	**	0.002	31.22	26.28	ns	0.498
95% CI lower	355.40	432.00			19.11	21.05		
95% CI upper	464.00	585.80			43.32	31.51		
Range	380.60	648.90			114.00	36.20		
	**MFI CD63**				**MFI CD81**			
	AM	PM	diff	p	AM	PM	diff	p
Mean	138.90	132.70	ns	0.444	49.53	57.23	*	0.0358
95% CI lower	103.30	99.78			30.77	37.38		
95% CI upper	174.50	165.70			68.28	76.78		
Range	275.50	273.60			130.60	146.50		

Figure S5. Flow cytometry analysis investigating diurnal impact on EV marker abundances. (A-D) EV surface marker expression presented as MFI in serum EVs from fed and fasted subjects (n=20). Statistical analysis used paired t-test, Error bars represent mean with 95% CI. (E) Summary data table of A-D.

Additional notes: EV samples from day 1 and day 3 were grouped (AM and PM, hence n=20).

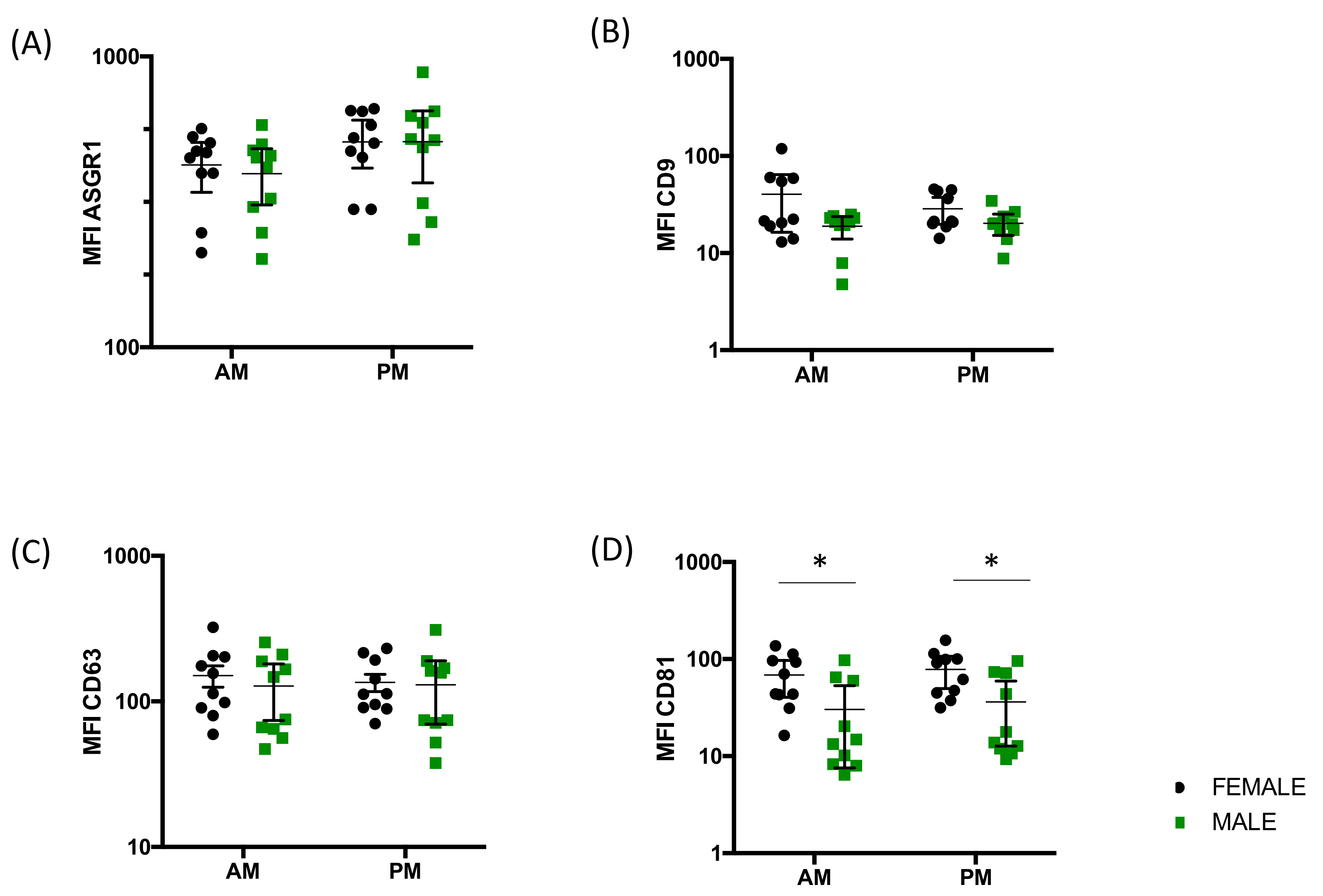

(E)

	**MFI ASGR1**							
	AM				PM			
	FEMALE	MALE	diff	p	FEMALE	MALE	diff	p
Mean	424.00	395.40			508.90	509.00		
95% CI lower	341.40	413.10	ns	0.972	413.10	367.60	ns	>0.999
95% CI upper	506.60	481.90			604.70	650.30		
Range	353.40	380.60			362.50	648.90		
	**MFI CD9**							
	AM				PM			
	FEMALE	MALE	diff	p	FEMALE	MALE	diff	p
Mean	40.28	18.87			28.65	20.22		
95% CI lower	16.35	13.94	ns	0.205	19.79	15.20	ns	0.203
95% CI upper	64.21	23.80			37.51	25.24		
Range	105.80	20.30			31.40	25.50		
	**MFI CD63**							
	AM				PM			
	FEMALE	MALE	diff	p	FEMALE	MALE	diff	p
Mean	150.40	127.50			135.30	130.20		
95% CI lower	93.37	73.98	ns	0.862	93.99	69.85	ns	0.998
95% CI upper	207.40	180.90			176.60	190.50		
Range	263.30	206.80			160.90	273.60		
	**MFI CD81**							
	AM				PM			
	FEMALE	MALE	diff	p	FEMALE	MALE	diff	p
Mean	68.68	30.37			78.36	36.10		
95% CI lower	40.45	7.55	*	0.038	49.57	12.68	*	0.029
95% CI upper	96.91	53.19			107.10	52.52		
Range	120.60	91.00			124.20	86.70		

Figure S6. Flow cytometry analysis investigating impact of sex on EV marker abundance. (A-D) EV surface marker expression presented as MFI in serum EVs males and females (n=10). Statistical analysis used one way ANOVA, Error bars represent mean with 95% CI. (E) Summary data table of A-D.
