# Supplementary material for: Importance of between and within Subject Variability in Extracellular Vesicle Abundance and Cargo when Performing Biomarker Analyses
Source: Cells. 2021 Feb 24;10(3):485. doi: 10.3390/cells10030485 (PMC7996254; doi:10.3390/cells10030485)
Supplement: Supplementary file 1 [file cells-10-00485-s001.zip › Supplementary data/Supplementary Table.pdf]

Table S1. Peptide sequences.

Analyte	Sequence
CD9	H2N-DVLETFTVK-OH
CD63	H2N-NNHTASILDR-OH
CD81	H2N-QFYDQALQQAVVDDDANNAK-OH
CANX	H2N-IVDDWANDGWGLK-OH
ALB	H2N-LDELRDEGK-OH
TSG101	H2N-VDANGK-OH
ASGR1	H2N-SLESQLEK-OH
